# The Electoral Coalition of the Radical Right in Western Europe

**DOI:** 10.1111/1468-4446.70062

**Published:** 2025-12-07

**Authors:** Florian Buchmayr

**Affiliations:** ^1^ Institute of Sociology University of Bremen Bremen Germany

## Abstract

While most research on the radical right attempts to identify the one central voting motive among its supporters, few studies have sought to differentiate between different types of voters. Given this research gap, we assume that there are multiple paths to the radical right and that different groups have different motives for their support for this party family. Based on different waves of the ESS, we conduct a cluster analysis in order to classify the ideological heterogeneity within the electorates of 15 Western European radical right parties across three conflict dimensions (redistribution, cultural liberalism, migration). We distinguish between four types of voters, analyse their social characteristics and try to identify different voting motives, ranging from defending economic status hierarchies to processing economic insecurities or protesting the loss of cultural hegemony. On the basis of these findings, we discuss what holds the electoral coalition of the radical right together and what can potentially divide it.

## 1 Introduction

The growing electoral success of the radical right (Hall et al. 2023, 2; Danieli et al. 2022, 40) has been accompanied by an increase in academic research on this party family (Hunger and Paxton 2022). There is a broad consensus that the radical right is particularly successful in appealing to individuals with low formal education and the working class (Lubbers et al. 2002; Ivarsflaten 2005; Oesch 2008; Bornschier and Kriesi 2012; Oesch 2012; Oesch and Rennwald 2018). More controversial, however, is the question of whether these findings indicate that the success of the radical right can be interpreted as an economic or a cultural phenomenon. On the one hand, there are those who see the overrepresentation of individuals with low formal education and members of the working class as a revolt by the losers of recent economic transformations (Betz and Betz 1994; Bornschier 2010; Fraser 2017). Members of the working class—a group that has traditionally voted for social democratic parties—are increasingly faced with bleak future prospects, making them more open to the radical right's promises of fundamental change. Indeed, there is evidence that the ability of welfare states to reduce economic risks has an impact on the likelihood of voting for radical right parties (Vlandas and Halikiopoulou 2022). On the other hand, there are studies that see the rise of right‐wing populism primarily as the result of a cultural backlash, as cultural attitudes, especially attitudes towards migration, have the greatest impact on the likelihood of voting for the radical right (Inglehart and Norris 2016; Bornschier and Kriesi 2012; Van der Brug et al. 2012; Sides et al. 2018; Oesch 2008), and the effects of social class often lose their significance when models control for these political orientations (Hartmann et al. 2022). Moreover, cultural anxieties about migration are a stronger driver of voting decisions than economic concerns about migration, such as worries about having to share limited resources with immigrants (Rydgren 2008; Lubbers and Güveli 2007; Lucassen and Lubbers 2012; Halikiopoulou and Vlandas 2020).

However, in the research literature, economic and cultural explanations are no longer seen as incompatible, as there are many studies highlighting the interconnectedness of cultural and economic motives. Numerous studies show that status anxieties, which can arise from both economic and cultural sources, are an important driver of support for the radical right (Bukodi and Goldthorpe 2021; Burgoon et al. 2019; Gest et al. 2018). With the transition to a post‐industrial knowledge society, the male industrial worker ‒ who embodied a social ideal and role model during the Fordist era ‒ has experienced a loss of status (Gidron and Hall 2017). Voting for right‐wing populist parties serves as a protest against both economic decline and the cultural devaluation of one's own way of life, which is overshadowed by the increasing hegemony of alternative lifestyles promoted by the new cultural middle classes (Reckwitz 2021). Thus, status anxieties serve as a bridge between economic and cultural explanations, as they encompass both economic concerns as well as perceptions of the symbolic legitimacy of one's cultural way of life. Another strand of research suggests that perceptions of economic conditions are strongly mediated by spatial milieus that cultivate specific interpretations of social reality. Economic developments in the immediate environment can trigger anxieties and concerns and cultivate specific scripts for interpreting social reality that affect residents regardless of individual experiences of economic deprivation (Adler and Ansell 2020; Manow 2018, 97; Fetzer 2019; Cramer 2016; Huijsmans 2022). From this perspective, cultural and economic explanations cannot be separated.

This brief review shows that the current literature manages to present complex motives for voting for the radical right, reflecting both economic and cultural factors. However, most studies try to identify one particular motive for voting for the radical right. While this perspective is undoubtedly important, there is comparatively little research on the extent to which different types of motivation exist and vary between different groups within the diverse radical right electorate. In what follows, we attempt to explore this perspective in greater depth, focussing on the heterogeneity of political attitudes. Using different waves of the ESS, we conduct cluster analyses based on three central conflict dimensions (redistribution, cultural liberalism and migration) and distinguish four types of voters. We discuss possible motives and causes for their affinity to radical right parties and reflect on how the radical right manages to mobilise its heterogeneous electoral coalition.

## 2 Multiple Paths to the Radical Right

While it is important to identify social or political characteristics that are over‐represented in the radical right's electorate, we should not lose sight of the heterogeneity of this group. There are a number of findings in this regard, most notably the fact that the radical right is popular among members of both the working class and the petty bourgeoisie (H. Kitschelt 1995; Ivarsflaten 2005; Oesch 2008; Ivarsflaten and Stubager 2012). In his qualitative study, Damhuis distinguishes three types of right‐wing thinking in France and the Netherlands, corresponding to different class positions. In a materially deprived working class, there is outrage at the unjustified privileges of migrants in contrast to poor, decent natives (Damhuis 2020, 118), while among more privileged class fractions (middle managers, salespeople or small business owners) a strict work ethic is upheld and migrants are seen as lazy beneficiaries of a broken system (Damhuis 2020, 141). In an educated upper middle class, economic motives are not important at all, as they are focussed on preserving the national cultural heritage, especially against the perceived threat of Islamisation, which is seen as culturally incompatible with their own society (Damhuis 2020, 165). This typology of voters by Damhuis is the only one of its kind. There are also several studies that indirectly shed light on the different motives of different classes by calculating different interaction effects. Oesch finds that fearing wage dumping caused by migrants is more important for the working class than for other classes (Oesch 2008), while Lucassen & Lubbers find the opposite (Lucassen and Lubbers 2012). Moreover, feelings of status loss are a stronger predictor of voting for right‐wing populist parties in the upper classes (Sachweh 2020), while relative income loss (Hartmann et al. 2022) or downward mobility compared to one's parents (Kurer and Van Staalduinen 2022) are more important for developing preferences for the radical right in the lower classes. Beyond class‐specific effects, there are also different motivations to vote for the radical right between individuals from urban/rural places (Harteveld et al. 2022) or different regions (Manow and Schwander 2022).^1^ Building on these studies, this paper focuses on the ideological heterogeneity of the electorate of the radical right in Western Europe. The following sections provide a definition of ideological heterogeneity (Section 2.1), review the current state of research regarding the political attitudes of voters of the radical right (Section 2.2), and present an approach for analyzing the ideological heterogeneity of an electorate by drawing on the concept of electoral equifinality (Section 2.3).

### 2.1 Analysing Ideological Heterogeneity

Following Federico (2019), we conceptualise ideological thinking as consisting of three characteristics. (1) Ideological thinking contains statements about both the current state of society and the desirable state to which it should aspire. These beliefs can be expressed in the language of major classical theories of political thought, as well as in the form of common‐sense and habitualised moral intuitions that may differ significantly from these theories (see, e.g., Freeden et al. 2015; Damhuis and Westheuser 2024). (2) Ideological thinking is organised into belief systems that employ a particular logic to connect attitudes from various dimensions of conflict. Here, we do not assume that certain combinations of attitudes are illogical and erratic rather than ideological (Converse 2006, 29 ff.). However, it is not uncommon for individuals to lack ‘genuine’ opinions on certain political issues (Bourdieu 1984, 397 ff.), which means that working with quantitative data runs the risk of assuming the presence of more ideological beliefs than actually exist. Furthermore, the reasons for connecting different positions cannot be directly observed in quantitative analyses, since it is not possible to directly ask respondents how they reconcile different positions regarding different conflict dimensions. (3) Ideological belief systems are socially embedded. This suggests that political attitudes are not formed spontaneously by individuals. Instead, they are generated and disseminated by social groups that heavily influence their members. Recent studies have shown that political attitudes are closely associated with social identities (Bornschier et al. 2021; Zollinger 2024), habitualised lifestyles (Purhonen and Heikkilä 2017; Jarness et al. 2019; Flemmen et al. 2022), and practices of symbolic boundary‐making (Jarness and Flemmen 2019; Westheuser and Zollinger 2025). These findings demonstrate that political attitudes play a significant role in both fostering cohesion within social groups and generating tension and friction between them.

In line with findings from attitude research, three central dimensions of attitudes can be distinguished that structure political conflict in Western Europe: redistribution, migration, and attitudes towards individual civil liberties (abortion, women's rights, homosexuality, drug use, etc.), which will be referred to in the following as “cultural liberalism” (see, e.g., Layman and Carsey 2002; Bornschier and Kriesi 2012; Laméris et al. 2018; Alexandre et al. 2021). While important areas of conflict are covered, the radical right also uses other issues to mobilise voters. Mudde (2019) identifies four topics that are of particular importance: (1) migration, (2) security and law and order, (3) corrupt elites, and (4) rejection of global solidarity and transnational institutions. While migration is considered directly, law and order issues are considered indirectly, as cultural liberalism measures the extent to which deviant behaviour should be punished, while traditional conformity is demanded. Criticism of corrupt elites and transnational organisations is not included in the cluster analyses. Attitude research shows that views on transnational organisations such as the EU are strongly correlated with anti‐migration views (see, e.g., Laméris et al. 2018). Given this strong correlation, including these items would not provide any additional information for the cluster analysis. Attitudes towards political elites were not considered because they do not represent ideological positions in the narrow sense (see Mudde 2017, 30), but are rather context‐dependent evaluations of the political system. Haugsgjerd (2019), for example, demonstrates that mistrust of political elites among radical right supporters decreases when their party is in government. Furthermore, although the ESS includes items on popular sovereignty and trust in political elites, it lacks items that adequately capture the moral dimension of radical right criticism of elites, which pits an idealised ‘pure people’ against a corrupt elite (Mudde 2017, 30). For these reasons, the following analyses are limited to the three attitude dimensions of redistribution, cultural liberalism, and migration.

### 2.2 Political Attitudes and the Radical Right

In the following we briefly summarise previous findings on the relationship between these conflict dimensions and the likelihood of voting for the radical right.

1. Migration: Voters of right‐wing parties seem to be primarily characterised by their anti‐immigration attitudes, and all studies find a strong positive effect of anti‐immigration attitudes on voting for right‐wing populist parties (Oesch 2008; Arzheimer 2008; Bornschier and Kriesi 2012; Ivarsflaten and Stubager 2012; Zhirkov 2014; Werts et al. 2013; Cavallaro and Zanetti 2020). However, studies also show that at least a third of voters have moderate or progressive attitudes (Stockemer et al. 2021), that not all aspects of migration are equally opposed (Halikiopoulou and Vlandas 2020), and that sometimes conservative attitudes towards cultural liberalism are sufficient to motivate people to vote for the radical right (Christley 2022).
2. Redistribution: In terms of economic attitudes, the radical right can mobilise individuals with both economically conservative and economically progressive views (Gidron 2022, 155 f., Damhuis 2020, 111 f.). This situation seems to be related to the socio‐structural heterogeneity of the radical right, as the two traditional core constituencies of the radical right ‐ manual workers and small business owners ‐ hold diametrically opposed economic preferences (Ivarsflaten 2005). It is therefore not surprising that radical right parties tend to keep their positions on the economic conflict dimension as ambiguous as possible (Rovny 2013; Rovny and Polk 2020). As the electorate of these parties is increasingly coming from the working class, they have shifted slightly to the left for strategic reasons (Afonso and Rennwald 2018; Eger and Valdez 2015; Rovny and Polk 2020), but remain as ambivalent as possible, often combining neoliberal economic policies with pro‐social rhetoric (Havertz 2020).^2^ Some studies find a weak positive effect of economically left‐wing attitudes (Van der Brug et al. 2012), others find a weak negative effect of economically left‐wing attitudes, which remains far below the explanatory power of cultural attitudes (Bornschier and Kriesi 2012; Ivarsflaten and Stubager 2012; Zhirkov 2014; Sipma et al. 2023), while other studies cannot find any significant effects at all (Ivarsflaten 2008; Attewell 2021, 2022; Cavallaro and Zanetti 2020; Arzheimer 2008). Part of this heterogeneity is due to the fact that the effects of redistributive stances differ across countries, as they seem to depend to some extent on the specific positioning of different right populist parties (Eger and Valdez 2015; Mazzoleni and Ivaldi 2020).
3. Cultural Liberalism: For the current wave of right‐wing populist parties, attitudes towards individual civil liberties are of secondary importance compared to migration (Arzheimer 2018), and there is considerable variation on this conflict dimension within the radical right. While some studies find no significant effect (Zhirkov 2014; De Koster et al. 2014), others find a positive effect of conservative attitudes towards cultural liberalism (Bornschier and Kriesi 2012; Christley 2022). Lancaster shows that there are large differences within the electorate of the Dutch PVV, with young voters in particular having relatively moderate or progressive attitudes on issues such as gender or sexuality (Lancaster 2020). This is not surprising given that some parties, such as the PVV, seek to instrumentalise their ‘modern’ views in their fight against a supposedly backward, anti‐modern Islam (Akkerman 2015).

### 2.3 The Concept of Electoral Equifinality

So far, research has mainly tried to locate the average radical right voter ideologically, with little effort to identify different types of right‐wing thinking. The attitudinal heterogeneity within the radical right electorate, particularly with regard to redistribution and cultural liberalism, indicates the presence of different ideological subgroups. In order to examine this ideological heterogeneity, we will draw on Damhuis's (2020) concept of electoral equifinality. A central premise of this concept is the assumption of causal heterogeneity. This means that not all parts of a given population are influenced by the same causal factors. Instead, different sets of causal factors can lead to the same result. This is why identifying different causal paths is more important than identifying the average characteristics of a given population (Damhuis 2020, 34). Very different attitudes and social positions can lead people to vote for the same party — in our case, the radical right. However, electoral equifinality is not about identifying as many causal paths as possible. Rather, it is about developing ‘medium‐level categorisations’ that strike a balance between considering small, individual idiosyncrasies and making very broad generalisations (Damhuis 2020, 35). The causal paths themselves should not be viewed as the outcome of different individual variables, but rather as the product of configurations of interacting factors (Damhuis 2020, 36). To describe the equivalent concept of electoral equifinality on the supply side of the political system, Damhuis (2020: 25) draws on Ernesto Laclau's theory of equivalence. According to this concept, political elites establish equivalences between very different individuals by abstracting from their heterogeneity and creating commonalities by dissociating them from other groups. The left, for example, tries to mobilise different class fractions by antagonising economic elites (Mouffe 2018). According to this view, political elites are not merely representatives of specific social groups; they also have considerable freedom to form their own electoral coalitions (Enyedi 2005). For Damhuis (2020: 26), the electoral coalition of the radical right is largely held together by nativism. This involves a demarcation both downwards against lazy and undeserving migrants as well as upwards, towards unpatriotic and treacherous elites who conspire against the ordinary people. Another way of describing the formation of electoral coalitions is the concept of ‘multivocality’. According to this concept, political elites address and mobilise several social groups by speaking multiple languages simultaneously (Gidron 2016, 30 ff.). From this perspective, the differences between various groups of the electorate remain intact and are not resolved through the discursive construction of equivalences. However, rather than focussing on the strategies employed by political elites to form electoral coalitions, the following analyses will examine different voting motives on the demand side of the political system. Cluster analysis will be used to identify different ideological groups within the radical right, while multivariate regression analysis will determine the social positions of these groups. Instead of focussing on the average characteristics of the electorate, analysing various combinations of attitudes will enable us to identify different paths that lead to support for the radical right.

## 3 Data and Methods

Our analyses are based on data from different waves of the European Social Survey (ESS 8 ‒ ESS 10). The survey dates of these waves took place between August 2016 (start of wave 8) and May 2022 (end of wave 10). This limits the analysis to the period in which the radical right reached its current electoral strength in the wake of the so‐called ‘refugee crisis’ (Mudde 2022). Table 1 lists all right‐wing populist parties included in the following analyses, with a total of 15 parties from 13 countries. For the purpose of the analyses, the radical right electorate is defined as those individuals who indicated they voted for one of these parties in the last national election in their respective countries.

**TABLE 1:** Overview of the parties included in the analyses.

Country	Party	ESS 8	ESS 9	ESS 10	ESS 8‐ESS10	Penultimate election	Last election
Austria	Freiheitliche Partei Österreichs Freedom party (FPÖ)	250 (15.70%)	301 (15.08%)	121 (7.15%)	672 (12.73%)	2019: 16.17%	2024: 28.85%
Belgium	Vlaams Belang Flemish interest (VB)	30 (2.16%)	28 (2.08%)	80 (7.67%)	138 (3.65%)	2019: 11.95%	2024: 13.77%
Switzerland	Schweizerische Volkspartei Swiss People's party (SVP)	148 (17.43%)	147 (23.19%)	166 (23.28%)	463 (21.08%)	2019: 25.6%	2023: 27.9%
Germany	Alternative für Deutschland Alternative for Germany (AfD)	58 (2.74%)	111 (6.82%)	328 (5.11%)	497 (4.39%)	2021: 10.4%	2025: 20.8%
France	Rassemblement National National Rally (RN)	125 (10.43%)	103 (10.98%)	107 (12.24%)	335 (11.47%)	2022: 17.3%	2024: 32.05%
United Kingdom	UK Independence party (UKIP)	108 (7.54%)	49 (3.18%)	/	157 (5.27%)	2019: 0.07%	2024: Reform UK—14.3%
Netherlands	Partij voor de Vrijheid Party for freedom (PVV)	102 (8.08%)	82 (6.80%)	73 (6.14%)	257 (6.75%)	2023: 23.49%	2025: 16.66%
Netherlands	Forum voor Democratie Forum for democracy (FvD)	/	18 (1.49%)	38 (3.20%)	56 (1.16%)	2023: 2.23%	2025: 4.54%
Denmark	Dansk Folkeparti Danish People's party (DF)	/	172 (13.98%)	/	172 (13.98%)	2019: 8.74%	2022: 2.63%
Finland	Perussuomalaiset True finns (PS)	193 (10.03%)	135 (11.20%)	133 (11.96%)	461 (11.11%)	2019: 17.5%	2023: 20.1%
Norway	Fremskrittspartiet Progress party (FrP)	121 (10.07%)	102 (9.26%)	89 (8.33%)	312 (9.26%)	2021: 11.7%	2025: 23.85%
Sweden	Sverigedemokraterna Sweden democrats (SD)	94 (6.92%)	141 (10.75%)	229 (12.13%)	464 (9.84%)	2018: 17.5%	2022: 20.5%
Spain	Vox Latin for ‘voice’	/	104 (10.58%)	104 (6.59%)	208 (8.26%)	2019: 15.1%	2023: 12.4%
Italy	Lega Nord Northern league (LN)	81 (4.81%)	265 (20.67%)	155 (14.38%)	501 (12.39%)	2018: 17.35%	2022: 8.77%
Italy	Fratelli d'Italia Brothers of Italy (FdI)	31 (1.84%)	38 (2.96%)	111 (10.30%)	180 (4.45%)	2018: 4.35%	2022: 25.9%

The following analyses focus on Western Europe, since voting for the radical right is driven by different factors in Eastern Europe. There are several reasons for this. Firstly, while parties in Western Europe tend to be organised along a left/right dichotomy, those in Eastern Europe follow the opposite logic. Communism in Eastern European countries forged a connection between economic protection and cultural conservatism. In the 1990s, this configuration became the central reference point for parties in the new, emerging party landscape. During the transition period, all supporters of neoliberal reforms also became advocates of democratisation and cultural liberalism, while the opponents of these reforms developed a culturally conservative profile (Vachudova and Hooghe 2009). Consequently, economically progressive parties tend to adopt culturally conservative views, while economically conservative parties tend to adopt culturally progressive views (Marks et al. 2006). Therefore, parties of the radical right are more economically progressive in Eastern Europe than in Western Europe. 2) Not only does electoral competition follow a different logic in Eastern European party systems, but so do individuals' combinations of political attitudes. A combination of economically progressive and culturally conservative attitudes is far more prevalent in Eastern Europe than in Western Europe (Malka and Soto 2019; Calzada et al. 2014, 3) The motives behind voting for radical right‐wing parties differ between Eastern Europe and Western Europe. Migration plays a greater role in Western Europe than in Eastern Europe, where societies have been more strongly influenced by emigration than immigration, and where the problematisation of national minorities (Roma, Turks in Bulgaria, Hungarians in Slovakia, etc.) has been particularly important for the success of right‐wing mobilisation (Minkenberg 2017; Kende and Krekó 2020; Bustikova and Kitschelt 2009). While there is evidence that the salience of these conflict dimensions has increased in recent years (Lancaster 2022), cultural liberalism remains more salient in Eastern Europe, which is also reflected in the fact that religiosity—one of the most important predictors of attitudes towards cultural liberalism—is a strong, positive predictor of voting for these parties in Eastern Europe, while it doesn't play a significant role for voting decisions of Western European parties of the radical right (Allen 2017; Marcinkiewicz and Dassonneville 2021). Our own analyses confirm these findings: cultural liberalism is a significantly stronger predictor of voting for the radical right in Eastern Europe than in Western Europe, while migration is a weaker predictor. Furthermore, religious and low‐income individuals in Eastern Europe tend to vote for radical right‐wing parties, whereas this is not the case in Western Europe (see Figure A12 in the Appendix). Simply combining cases from Western and Eastern Europe is problematic, given the differences between these two regions. Including Eastern European cases would only be meaningful if the analysis aimed to compare the two regions, which would exceed the scope of this paper. This approach would require a second cluster analysis of Eastern European voters and a comparison of the cluster solutions and the social characteristics associated with the clusters. For this reason, the following analyses will focus on voters of Western European radical right‐wing parties.

The analysis consists of three steps: (1) distinguishing between different conflict dimensions (factor analysis) (2) constructing groups with similar attitudes (cluster analysis) (3) analysing the socio‐structural characteristics of the clusters (regression analysis).

### 3.1 Factor Analysis

Numerous studies have identified three attitude dimensions (redistribution, cultural liberalism, migration) as central conflict lines in Western Europe (see, e.g., Gidron 2016; Häusermann and Kriesi 2015; H. Kitschelt and Rehm 2014; Layman and Carsey 2002). These attitude dimensions also reflect the evolution of political conflicts in recent decades. Since the beginning of industrialisation, economic distributional conflicts have been the driving force of political competition, while cultural conflicts, largely of a religious nature, have been present, but have generally played a smaller role in political mobilisation than economic issues (Lipset 1959, 483). In the 1970s and 1980s, however, the nature of political competition underwent a fundamental transformation as cultural issues became increasingly prominent. In particular, ‘postmaterial values’ (Inglehart 1997) and issues regarding individual liberties (abortion, gender equality, drug use) were politicized during this period (H. Kitschelt 1994). In the 1990s, a third conflict dimension emerged, as the issue of migration became increasingly salient (Kriesi et al. 2006; Evans and Mellon 2019; Grande et al. 2019). The items covering these attitude dimensions (economic inequalities, cultural liberalism, migration) can be found in the Appendix (Supporting Information S1: Table A1 in the Appendix). As the inclusion of items measuring attitudes towards redistribution varies between waves, we have to use different items to operationalise this dimension of conflict. Research shows that the economic conflict dimension itself is multidimensional. Besides conflicts surrounding the redistribution of economic resources from top to bottom, attitudes towards the deservingness of social welfare recipients follow a distinct logic (Attewell 2021; Cavaillé and Trump 2015; Achterberg et al. 2011; Derks 2004, 2006). However, this second economic conflict dimension, concerning the legitimacy of welfare state benefits, appears to correlate with cultural conflicts to some extent (Cavaillé 2023). Individuals with xenophobic attitudes, for example, tend to imagine foreigners as lazy welfare recipients and therefore reject generous assistance for the poor. Our own analyses of ESS 8 data, which include questions on the willingness of social welfare recipients and unemployed individuals to work, confirm this pattern (Supporting Information S1: Table A7 in the Appendix). Deservingness items do not load on the redistribution factor, but they cross‐load on the migration factor. This is why we refrain from considering a second economic conflict dimension.

Attitudes towards homosexuality are used to operationalise cultural liberalism. Ideally, a variety of issues, such as attitudes towards gender inequalities, drug policies, abortion, and conformity with traditions, would be considered when analysing this attitude dimension (see, e.g., Layman and Carsey 2002; Bornschier and Kriesi 2012; Laméris et al. 2018; Alexandre et al. 2021). Due to data limitations, homosexuality attitudes serve as the primary indicator for this dimension. However, additional factor analyses of ESS 8 data, which include items on gender inequalities as well as the willingness to conform to traditions, confirm that all these items load onto one factor, demonstrating that attitudes towards homosexuality are a valid indicator of a broader conflict dimension regarding cultural liberalism (Supporting Information S1: Table A6 in the Appendix).

In attitude research, attitudes towards climate change are sometimes considered an independent dimension of political conflict (Kenny and Langsæther 2023; Mau et al. 2023). Furthermore, this attitude dimension gained salience in recent years, particularly as the radical right increasingly mobilises voters by embracing extremely conservative stances on climate change (Dickson and Hobolt 2025; Schwörer and Fernández‐García 2024). However, this conflict dimension was not included in the analyses because the ninth wave of the ESS did not include any items on this topic. To ensure that omitting this dimension did not affect the identification of different voter groups, cluster analyses were performed using climate change attitudes alongside attitudes towards redistribution, cultural liberalism and migration (Supporting Information S1: Tables A4 & A5 and Figure A11 in the Appendix). There are no fundamental differences between the cluster structures of the analyses with and without climate attitudes, suggesting that omitting climate change attitudes had no significant impact on the results presented in this paper.

The factor analyses were carried out with orthogonal rotation and separately for all 13 countries and for all respondents in each country. Otherwise, the cluster analyses would reflect country‐specific differences in the level of progressiveness. Progressive redistributive attitudes are more prevalent in southern Europe, while northern European countries are on average more culturally progressive (Wulfgramm and Starke 2017, 11; Gusciute et al. 2021, 158). As a result, economically more progressive and culturally more conservative clusters could, for example, be largely composed of respondents from Southern Europe. However, the aim of the present analysis is to look at the differences between radical right voters and other voters in their respective countries. As an additional validation of the multidimensionality of political attitudes in the radical right electorate, a factor analysis was also conducted with members of this group only. The results confirm the assumption that we can distinguish between the three attitude dimensions described above (Supporting Information S1: table A2 in the appendix).

### 3.2 Cluster Analysis

Three different clustering methods are used and combined for the analysis. Firstly, the single‐linkage method is used to identify outliers, which are then excluded from the analysis. Secondly, Ward's method, a hierarchical clustering method, is used to create an initial cluster structure, which serves as a starting point for the final K‐means clustering, where an optimised allocation of cases takes place. Two criteria are used to decide how many clusters to generate. On the one hand, the Calinksi‐Harabasz index, which indicates the degree of internal homogeneity and external heterogeneity of different cluster solutions. On the other hand, the plausibility of different cluster solutions has to be assessed. To validate the final cluster structure, we run alternative clustering methods or sample the population before running the cluster analysis again. If these methods produce similar results, we can assume that the identified cluster structure is reasonably robust.

### 3.3 Multivariate Analysis

Once the clusters have been identified, their social characteristics are examined in more detail. We focus on different indicators of class positions and consider education and income to be particularly relevant (Bourdieu 1984). Furthermore, results from attitude research demonstrate that education and income are key determinants of political attitudes, with higher levels of education positively predicting culturally progressive attitudes, while higher levels of income negatively predict economically progressive attitudes (Svallfors 2006; Häusermann and Kriesi 2015; Lindh and McCall 2020). Household income is used to construct income classes, adjusting for differences in household size. Respondents are assigned to one of three groups: lower class (up to 75% of the median income), middle class (between 75% and 150% of the median income), and upper class (above 150% of the median income). Since income is measured in categories in the ESS, individuals are assigned the mean values of the different cut‐off points. For the open‐ended income category of the largest income group, the procedure proposed by Hout is used to determine a cut‐off point (Hout 2004). With respect to educational attainment, respondents are classified into one of three groups: individuals with no secondary education, individuals with secondary education but no tertiary education, and individuals with tertiary education. Another indicator of class position is provided by occupational class schemes. Cleavage research shows that, in recent decades, the industrial working class has increasingly turned away from left‐wing parties and become a core constituency of the radical right (Oesch and Rennwald 2018). As occupational positions are strongly correlated with education and income, we examine their effects separately from those of education and income (Lindh and McCall 2020, 283; H. P. Kitschelt and Rehm 2023, 524 ff.). For this purpose, we employ Oesch's (2006) class scheme, which emphasises horizontal differentiation between various work logics and distinguishes, for example, between an industrial working class and a service proletariat.

In addition, several other characteristics known to be potential determinants of political attitudes are included: gender, age, religiosity, and place of residence (urban vs. rural).

## 4 Ideological Differences Within the Radical Right

**FIGURE 1:**
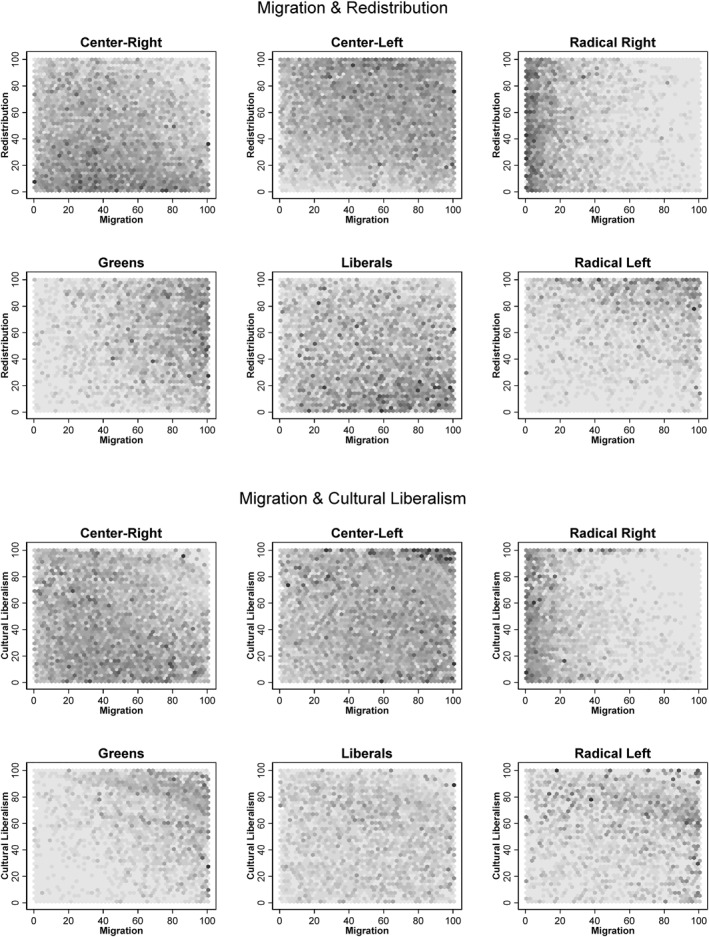
Hexplots for voters from different party families. The top part shows the overlap between economic attitudes and attitudes towards migration, the bottom part shows the overlap between cultural liberalism and migration. All conflict dimensions have been recoded so that high values indicate conservative attitudes. The darker the fields, the higher the concentration of voters.

Before identifying different ideological groups within the electorate of the radical right, we take a closer look at the general attitudinal differences within this group. Figure 1 presents hexplots showing the density of voters from different party families in a two‐dimensional space (redistribution and migration or cultural liberalism and migration). The attitude dimensions have been recoded to range between 1 and 100, with higher values representing progressive and lower values conservative positions. The darker the squares, the higher the concentration of voters from a particular party family. A clear pattern emerges. While attitudes towards migrants tend to be hostile, attitudes towards redistribution and, to a lesser extent, cultural liberalism vary considerably. These results are consistent with previous findings concerning the political attitudes of radical right‐wing voters (for economic attitudes, see Gidron 2022, 155 f., Damhuis 2020, 111 f.; for cultural liberalism, see Lancaster 2020).

**FIGURE 2:**
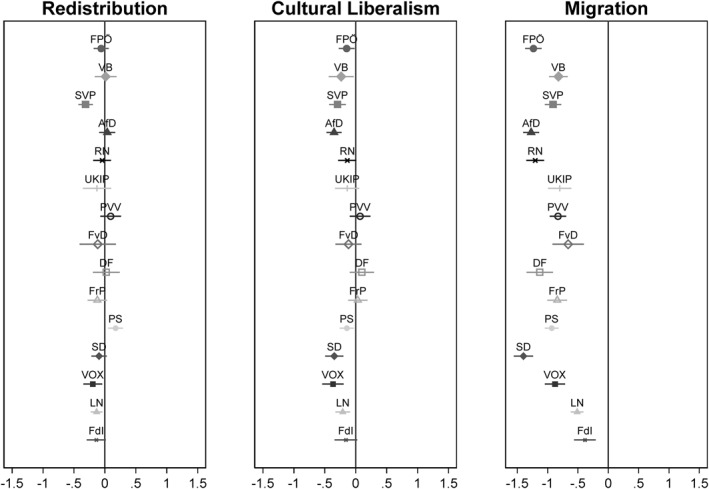
Coefficient plot showing the effects of different attitude dimensions on the likelihood of voting for different radical right parties; based on logistic regressions controlling for gender, age, city/country, religiosity and the other two attitudinal dimensions. All analyses include survey weights.

However, there are certainly some relevant differences between the 15 parties. The coefficient plot in Figure 2 shows the effects of different conflict dimensions on the probability of voting for a particular radical right party. For this purpose, 15 logistic regressions were carried out, with the dependent variable indicating whether the party in question was voted for or not. The three central conflict dimensions (redistribution, cultural liberalism, migration) were included as key independent variables, while also controlling for gender, age, urban/rural and religiosity. As income and education are the main determinants of economic and cultural attitudes (Svallfors 2006; Häusermann and Kriesi 2015; Lindh and McCall 2020), these two variables were not included in the models presented here. However, additional models that also control for these status variables show that they do not cause major shifts (Supporting Information S1: Figure A1 in the appendix). All attitudinal dimensions have been recoded so that high values represent progressive attitudes. The effects were also calculated by country rather than by party, since there are two countries (Italy and the Netherlands) with two parties of the radical right (Supporting Information S1: Figure A9 in the Appendix).

While anti‐immigration attitudes significantly increase the likelihood of voting for all 15 parties, the picture is more mixed for the other two conflict dimensions. Attitudes towards redistribution do not matter for most parties. There is a slight tendency for progressive economic attitudes to have a negative effect for most parties, but these effects are only significant for three parties (SVP, Vox, LN). In contrast, a significant positive effect of progressive economic attitudes is only found for one party (PS). In the case of the conflict dimension of cultural liberalism, there is a negative effect of progressive attitudes for most parties, which is significant for eight parties (FPÖ, VB, SVP, AfD, PS, SD, Vox, LN), although the strength of the effects is significantly lower than in the case of migration attitudes. Overall, we see that the radical right primarily attracts people with anti‐migration attitudes, while attitudes towards cultural liberalism and, in particular, redistribution are less clear‐cut. However, this does not mean that these attitudes do not influence voters' decisions; the results merely provide insight into the diversity of political attitudes among the radical right's electorate. It is possible that economically progressive attitudes influence the voting decisions of some voters, while economically conservative attitudes matter for others. Following the concept of electoral equifinality, it is necessary to abandon the implicit assumption of causal homogeneity and the focus on the average characteristics of the electorate, in favour of a more differentiated, typological approach that highlights the various ideological belief systems that lead to support for the radical right. The following chapter will use cluster analyses to illustrate and systematise this heterogeneity within the radical right in Western Europe.

## 5 Cluster Analysis

### 5.1 Construction of Clusters

In order to determine the appropriate number of clusters, the Calinski‐Harabasz index was used, which provides measures of the distinctiveness of different cluster solutions (Supporting Information S1: Table A3 in the appendix). As the 4‐cluster solution is the most distinctive according to this index and shows a high degree of plausibility and interpretability, the following analyses will focus on it. The results of the other cluster solutions can be seen in Figure A2 in the appendix.

**FIGURE 3:**
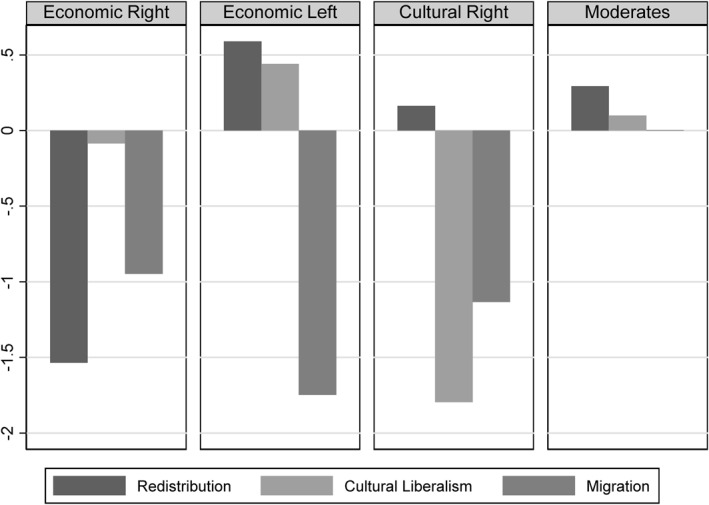
Illustration of the political differences between the groups of the 4‐cluster solution. Cluster Analysis calculated with voters from 15 parties on the basis of country‐specific factors using data from the ESS 8—ESS 10.

**FIGURE 4:**
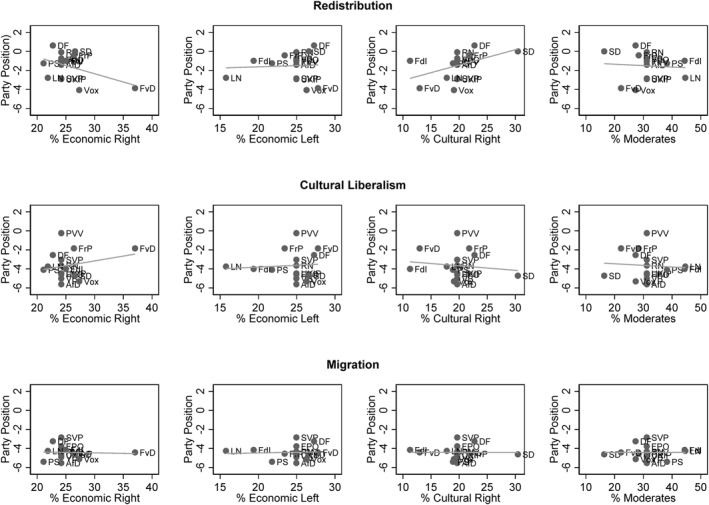
Scatterplots showing the relationship between cluster sizes and party positions regarding redistribution, cultural liberalism and migration. Party positions are calculated using CHES 2019 data and centred around country‐specific averages.

Figure 3 shows the results of the cluster analysis. The four groups are approximately equal in size: Economic Right 24.19%, Economic Left 24.96%, Cultural Right 19.65% and Moderates 31.19%. In addition to anti‐immigration attitudes, the Economic Right is characterised by strongly conservative economic attitudes, while the average positions on the conflict axis of cultural liberalism are moderate. Inequalities appear to be legitimate in principle, as long as they do not restrict personal freedoms. The Economic Left is characterised by a combination of anti‐immigration and pro‐distribution attitudes. This group insists on the creation of economic equality, but only among natives. The Cultural Right differs from this group in that it also has very conservative attitudes towards cultural liberalism. Although the members of this cluster support a degree of economic equality, they yearn for a completely culturally homogeneous society in which all forms of deviant behaviour are punished. The last cluster shows comparatively moderate attitudes in all three conflict dimensions and has been named accordingly. To test the stability of this cluster solution, several robustness checks were performed. We investigated whether a cluster analysis based on the Ward method alone, rather than in combination with the K‐means procedure, would produce similar results, which is absolutely the case (Supporting Information S1: Figure A3 in the appendix). In addition, 1000 cluster analyses were performed without a hierarchical clustering procedure, using only the K‐means procedure, starting clustering from arbitrary points. To check whether this leads to systematically different results, we use the Adjusted Rand Index, which ranges between 0 and 1 and indicates how similar the assignment of cases from two different cluster solutions is, with a value of 1 indicating exact agreement of classifications (Vinh et al. 2010). In the present case, comparisons of our cluster solution with the 1000 K‐means cluster analyses yield an average ARI of 0.966, indicating an extremely high degree of similarity. We also conducted a latent profile analysis, a probabilistic method of cluster analysis that assigns each respondent a probability of belonging to one of the constructed clusters. This clustering method produces partially different results. While three groups correspond to those of the other cluster solutions, there is no cluster of Moderates, but rather a group with progressive attitudes towards cultural liberalism (Supporting Information S1: figure A4 in the appendix). A group of Moderates starts to emerge, however, when a 6‐cluster solution is chosen, suggesting that the two clustering methods assign different levels of importance and prevalence to the group of Moderates. Another way to determine the robustness of the found cluster structure is to randomly sample the data before clustering and analyse whether these changes affect the results of the cluster analyses. We drew 200 random samples representing 95% of all cases and 200 random samples representing 50% of all cases and performed cluster analyses to compare their similarity to our final cluster solution. The average ARI values were 0.958 for the cluster solutions based on 95% of the cases and 0.918 for the cluster solutions based on 50% of the cases. Again, these results indicate a strong robustness of our final cluster solution. To make sure that the combination of different waves did not influence the result of the cluster structure, the cluster analysis was also carried out separately for each wave. The results hardly differ from the final cluster solution (Supporting Information S1: Figure A5 in the appendix). The cluster analyses were also carried out separately for each country. There are some countries that deviate from the cross‐country cluster solution, most notably Switzerland, but overall the results are very similar in most countries (Supporting Information S1: Figure A6 in the appendix). Finally, we examined whether the sizes of the clusters within the different electorates are correlated with the parties' positions on the different conflict dimensions. The distribution of clusters within parties is detailed in Supporting Information S1: Table A8 in the Appendix. Figure 4 plots these cluster sizes against the parties' positions on the three attitude dimensions, as measured in the CHES 2019 expert survey. High values on the *X*‐axis represent progressive party positions (compared to all other parties in a given country), while the *Y*‐axis depicts the sizes of the respective clusters. Overall, a party's policy profile has little influence on whether certain clusters are over‐ or under‐represented. For example, culturally liberal voter groups are not particularly overrepresented in parties that also hold comparatively liberal positions. Conversely, the Cultural Right, which holds extremely conservative views on this issue, is not overrepresented in parties that can directly cater to these preferences. Regarding parties' positions on migration, there is little variation, as all radical right parties occupy distinctly conservative positions compared to other parties in their respective countries. In the case of economic positions, there are some notable correlations. Parties of the radical right with economically conservative positions tend to have a larger share of voters from the Economic Right, while more economically progressive parties have on average a slightly larger share of voters from the Cultural Right. However, parties that are relatively progressive in terms of economic issues do not tend to have a larger proportion of voters from the Economic Left. This suggests that an economically progressive profile is not a prerequisite for attracting a significant proportion of economically progressive voters. Therefore, the positioning of radical right‐wing parties has little systematic influence on the sizes of the various voter groups.

### 5.2 Multivariate Analysis of the Clusters

**FIGURE 5:**
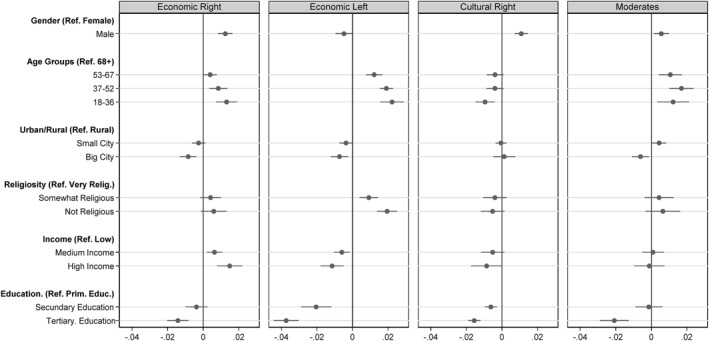
Coefficient plot of the influence of socio‐structural characteristics on cluster membership. Marginal effects based on multinomial regressions, controlling for country and ESS round. Standard errors clustered by country and year. Models include survey weights.

We then analysed the social characteristics of the four clusters. For this purpose, multinomial regressions were carried out in which the four clusters as well as individuals who indicated voting preferences beyond the radical right represent the five categories of the dependent variable. We calculated marginal effects and presented them graphically in the coefficient plot in Figure 5. In addition, these analyses were carried out separately for each of the 13 countries. Although many effects are not significant due to the smaller number of observations, there are very few cases where the effects go in opposite directions in different countries, underlining the robustness of our findings (Supporting Information S1: Figure A13 in the Appendix). Members of the Economic Right tend to be young, male and less educated, but have high income levels. Drawing on Bourdieu's model of competing class fractions, the Economic Right appears to be over‐represented in middle class fractions that have more economic than cultural capital. This is also supported by additional analyses showing that large employers are more likely to belong to this group, although this effect is not significant (Supporting Information S1: Figure A10 in the Appendix). This group finds itself in competition with cultural class fractions, that is, individuals who have more cultural than economic capital and are characterised by a modern, often cosmopolitan lifestyle (Bourdieu 1984, 114 ff.). These two class fractions struggle over the overall social significance of their respective types of capital and thus also over whether the relevance of cultural differences, for example with regard to different lifestyles, or economic inequalities should be accentuated (Lamont 1987, 1504; Flemmen 2014, 562). The Economic Right is therefore interested in maintaining existing economic privileges. Economic inequalities are perceived as legitimate because they are seen as the result of free market competition. Migrants can be rejected because they are perceived as weak market actors who cannot compete with the hard‐working native population. In his qualitative study, Damhuis also identifies a type of voter who may not be highly educated, but who takes his own (moderate) economic success as an invitation to demand a rigorous work ethic from others, and who is concerned that social norms of reciprocity are being violated by supposedly lazy migrants (Damhuis 2020, 141 ff.).

Young, rural and individuals with low formal education are over‐represented within the Economic Left. However, the main difference with the Economic Right is the low income level of this group. Furthermore, the group is characterised by an overrepresentation of both manual workers and service workers (Supporting Information S1: Figure A10 in the Appendix). This suggests that this cluster's sympathy for the radical right is linked to their economic insecurity. A large body of work hypothesises that, in the context of major structural changes, the working class is increasingly beginning to ethnicise economic inequalities. Most prominently, Eribon describes how parts of the working class have always had racist attitudes, but have now made them the central organising principle of their own political thinking (Eribon 2013, 143 f.). Migrants who ‘cut in line’ become a symbol of the unfulfilled promises of upward mobility in post‐Fordist societies (Hochschild 2016, 137 ff.), allowing the radical right to mobilise the losers of economic modernisation by resorting to a cultural language (Bornschier 2010, 200). Although the working class already exhibited authoritarian attitudes in earlier decades, the majority of them voted for labour parties (Lipset 1959). Now, however, economic conflicts seem to have lost salience in favour of cultural conflicts (De Vries et al. 2013), partly because social democratic parties have moved to the right (Evans and Tilley 2012), rendering the working class increasingly open to mobilisation efforts by the radical right (Spies 2013). Interestingly, women are overrepresented in this group, a finding also reported by Damhuis (2020: 120) for a similar voter type. One possible explanation is that this group is relatively liberal in terms of cultural liberalism, and that men are more inclined than women to support the radical right because of its promise to restore traditional gender relations (see, e.g., Sauer 2020).

Among the Cultural Right, individuals with low levels of formal education are overrepresented, while a negative income effect is not significant. The additional analyses of occupational classes show that manual workers are overrepresented and there is also a positive effect for small business owners, although this is not significant at the 0.05 level. This group differs from the other clusters in that older rather than younger age groups are overrepresented. The fact that this cluster also holds culturally conservative attitudes suggests that this group may be driven by cultural rather than economic insecurities. According to Norris and Inglehart's cultural backlash hypothesis, many cultural attitudes, especially those that can be attributed to the conflict dimension of cultural liberalism (gender equality, homosexuality, abortion, drug use), have gradually become more liberal in recent decades (Norris and Inglehart 2019). This is why older voters, in particular, protest against the loss of hegemony of their beliefs by voting for the radical right. Against this background, it comes as no surprise that, in contrast to the Economic Left, men are overrepresented.

Interpreting the motives of the fourth cluster poses a greater challenge. Moderates come from rural areas, are relatively young and have low levels of formal education ‒ social characteristics that are also predictors for most of the other clusters. Furthermore, as with the Economic Left and Cultural Right, manual workers are overrepresented here (Supporting Information S1: Figure A10 in the appendix). These results suggest that some of those group affiliations may provide a possible path to the radical right, even if individuals hold relatively moderate beliefs. In particular, the radical right is able to mobilise a coalition of individuals with low formal education, a finding supported by supplementary analyses showing that this group is over‐represented in almost all 15 parties ‒ even when we control for political attitudes, gender, urban/rural, religiosity, age, and income. Different income levels, on the other hand, do not seem to play a significant role (Supporting Information S1: Figure A7 in the appendix). The particular appeal of the radical right among individuals with low formal education may be due to the fact that this party explicitly appeals to this group by denigrating highbrow culture and celebrating its down‐to‐earthness in its political performances, creating an affective bond between the representative and the represented (Ostiguy 2017; Westheuser 2020). Thus, the dissociation from highbrow culture is not merely a question of aesthetic preferences. Those with lower levels of formal education feel culturally distant from political elites, whom they view as detached and unconcerned with their worries and concerns (Van Noord et al. 2023; Noordzij et al. 2019, 2021). These accusations are not entirely without substance, as the political preferences of individuals with lower levels of formal education are indeed given much less consideration by political elites than those of people with higher levels of formal education (Schakel and Van der Pas 2021).

On the other hand, it is also possible that the voting preferences of Moderates can be explained by political attitudes that were not taken into account for the cluster analyses. A look at the research literature shows that attitudes towards the political system are also identified as a key motivator for voters of the radical right (Kriesi and Schulte‐Closs 2020; Geurkink et al. 2020; Zhirkov 2014; Van der Brug et al. 2012), as strong criticism of political elites and the invocation of a suppressed popular will are central components of the radical right's communication (Mudde 2017). For this reason, the respondents' proximity/distance to the political system was analysed in additional models (Supporting Information S1: Figure A8 in the appendix). In contrast to the other clusters, trust in political institutions does not play a central role for this group, but they are also characterised by the feeling that outsiders have no influence on the decisions taken by the political system (External Political Efficacy) and express the wish that it is not the political elites but the will of the common people, which is imagined to be homogeneous, that dictates political decisions (Preference for Popular Sovereignty). Moderates thus show that the anti‐elitist rhetoric of the radical right makes them a viable alternative, even if potential voters do not have strong anti‐immigration attitudes. In light of the tendency of Moderates to distance themselves from the political system, it is important to bear in mind that individuals with limited political knowledge or interest often opt for middle‐of‐the‐road response categories as they lack clear positions on the issue at hand (Nadler et al. 2015; Sturgis et al. 2014). Therefore, it is possible that the supposedly moderate positions found in this cluster are more indicative of a lack of opinion than a neutral stance.

## 6 Conclusion

While much of the research literature on the radical right searches for a single cause of radical right voting preferences, this paper attempts to identify different types of radical right voters. Based on the concept of electoral equifinality, we assume that a phenomenon can have more than one cause and that very different paths can lead to the same outcome. The empirical analyses focussed on the ideological heterogeneity of the electorate and identified four different types of voters and their characteristics. Radical right voters with anti‐immigration and anti‐redistribution attitudes have low levels of formal education and high levels of income and seek to defend economic privileges (Economic Right). Individuals with anti‐immigration and pro‐redistribution attitudes, on the other hand, have low levels of both education and income and seem to deal with economic insecurities by culturalising economic and distributional conflicts (Economic Left). Individuals with regressive attitudes on both cultural conflict dimensions are older than the other clusters, and their voting preferences appear to be linked to a desire to restore cultural hegemony destroyed by cultural value change (Cultural Right). The last group holds relatively moderate attitudes, showing that the appeal of the radical right can extend beyond the narrower circle of individuals with decidedly anti‐immigration attitudes, as long as they have low levels of formal education and/or feel politically alienated (Moderates).

The proposed perspective of electoral equifinality is not intended to replace the conventional statistical analysis of the overrepresentation of certain characteristics within a party's electorate, but rather to complement it. While a more differentiated perspective is not necessarily superior, it offers a different view of the phenomenon and can enrich our understanding of party electorates. However, the more closely one zooms into party electorates, the greater the risk of becoming lost in the description of varieties, to the extent that one is no longer able to contribute anything substantial to the study of the phenomenon. Adopting a perspective of electoral equifinality therefore always involves striking a balance between abstraction and differentiation. The present analyses have shown that characteristics that are significantly overrepresented within the entire electorate should not be the only focus of interest; non‐significant results that point to heterogeneity and potential internal divisions may also be of interest and justify closer examination.

The results offer a potential bridge between economic and cultural explanations of voting for the radical right, as there may be groups where economic factors dominate and groups where cultural factors are prioritised. For the Economic Left, economic insecurities seem to play a greater role than in the case of the Cultural Right, where cultural insecurities appear to be more dominant. However, the identified motives of the different clusters must be treated with caution, as they represent interpretations based on political attitudes and social characteristics that need to be validated by qualitative research. The results of cluster analyses cannot speak for themselves and always require a fair amount of interpretation by the researcher. In this case, this involves making assumptions about the implicit logic of combining different attitude dimensions. To the best of our knowledge, Damhuis (2020) is the only qualitative study to date that identifies different groups of voters within the radical right‐wing electorate. Further qualitative studies could validate, extend or revise the presented results. Furthermore, it would be wrong to assume that only the voters of the radical right are heterogeneous. This is why it seems worthwhile to identify internal divisions within the electorates of other parties, too. For instance, there are many reasons to believe that green party voters mirror voters of the radical right, as there is a broad consensus regarding cultural conflicts, while economic attitudes seem to be more dispersed (see Figure 1).

It seems also worthwhile analysing different types of radical right voters in Eastern Europe. In this region, the structure of party systems (Marks et al. 2006), the salience of political conflict dimensions (Minkenberg 2017; Kende and Krekó 2020; Bustikova and Kitschelt 2009) as well as the historically grown loyalties between social groups and party families (Powell and Joshua 2014; Haughton and Deegan‐Krause 2015) differ greatly. Against this background, we can assume to find different types of voters of the radical right in Eastern Europe compared to those identified in Western Europe.

It is also important to bear in mind that we have ‘only’ focussed on three dimensions of conflict and that there may be other identities, perceptions or attitudes that could be key motivators for certain groups. Nevertheless, it is possible to make some assumptions about the electoral coalition of the radical right. A large part of the electorate seems to be held together by anti‐immigration attitudes, which can bridge major ideological differences on other dimensions of the conflict. This coalition may succeed for several reasons. On the one hand, voters of the radical right often seem to overestimate the degree of ideological agreement between themselves and their preferred party (Steiner and Hillen 2021), a misunderstanding that the parties actively encourage by framing their economic positions as vague and ambiguous as possible due to the strong ideological differences within their electorate (Rovny 2013). Another element enabling the emergence and stability of this coalition is the increasing salience of migration attitudes (Danieli et al. 2022; Bonikowski 2017) ‐ a reality that the radical right has actively helped to create (Williams and Hunger 2022). Given this high salience of migration, it is not surprising that radical right voters are willing to make major compromises as long as their anti‐immigration preferences are met (Kirkizh et al. 2023). At the same time, the Moderates demonstrate that the radical right electorate cannot be characterised solely as a coalition of anti‐immigration extremists. There is an alternative route to the radical right that does not involve xenophobic attitudes, and other factors may be responsible for holding the electoral coalition of the radical right together. While the level of economic capital varies widely, a low level of education seems to be a common denominator for very different sub‐groups of the radical right. The radical right actively tries to appeal to individuals with low levels of formal education by offering political performances that disparage high culture and celebrate their own down‐to‐earthness (Ostiguy 2017; Westheuser 2020). On the other hand, all sub‐groups of the radical right, including Moderates, share a sense of political alienation, which the radical right also explicitly addresses by presenting itself as an alternative to degenerate, decadent elites and as the voice of the common people (Mudde 2017).

Given these results, how can the current success of the radical right be mitigated? First, one option would seem to be to reduce the salience of the issue of migration and increase the salience of other dimensions of conflict that divide the radical right's electoral coalition. Similarly, after the Second World War, labour parties were able to attract authoritarian sections of the working class by keeping economic issues salient (Lipset 1959). The accommodation of anti‐immigration positions by mainstream parties, on the other hand, only serves to increase the salience of this dimension of the conflict (Abou‐Chadi and Krause 2020). Second, the appeal of the radical right to individuals with low formal education could be mitigated by directly targeting this group. Eribon highlights that the French working class has become alienated from left‐wing parties because they have made less and less effort to represent this group (Eribon 2013), and empirical studies also show that social groups respond positively when parties try to address them directly and explicitly (Robison et al. 2021).

Finally, other parties can contest the radical right's hold on voters with anti‐elitist attitudes. While the radical right's contempt for political elites is also linked to a contempt for democratic institutions, it is also possible to legitimately criticise political institutions and political elites. Political systems in Western European societies often have democratic deficits that are rarely politicised, such as the influence of lobbyists, the lack of responsiveness of political elites, or the framing of political decisions as being without alternative (Crouch 2008; Mair 2006). The mobilisation of political disenchantment and discontent should not be left to the radical right.

## Funding

The author has nothing to report.

## Conflicts of Interest

The author declares no conflicts of interest.

## Supporting information


Supporting Information S1

## Data Availability

ESS Round 10: European Social Survey (2023): ESS‐10 2020 Documentation Report. Edition 3.0. Bergen, European Social Survey Data Archive, Sikt ‐ Norwegian Agency for Shared Services in Education and Research, Norway for ESS ERIC. doi: 10.21338/NSD‐ESS10‐2020. ESS Round 9: European Social Survey (2021): ESS‐9 2018 Documentation Report. Edition 3.1. Bergen, European Social Survey Data Archive, Sikt ‐ Norwegian Agency for Shared Services in Education and Research, Norway for ESS ERIC. doi: 10.21338/NSD‐ESS9‐2018. ESS Round 8: European Social Survey (2020): ESS‐8 2016 Documentation Report. Edition 2.2. Bergen, European Social Survey Data Archive, Sikt ‐ Norwegian Agency for Shared Services in Education and Research, Norway for ESS ERIC. doi: 10.21338/NSD‐ESS8‐2016.

## Endnotes

^1^
Steiner et al. (2022) don't look at party preferences but show that distinct feelings of lacking recognition can trigger populist attitudes among distinct social groups.

^2^
A nuanced examination shows that the economic positions of the radical right vary considerably between different parties, but they are always characterised by strong welfare chauvinism, that is the exclusion of migrants from welfare state benefits (Ennser‐Jedenastik 2018; Otjes et al. 2018) and ‘producerism’, the rejection and stigmatisation of the ‘undeserving poor’ (Rathgeb 2020; Abts et al. 2021; Afonso and Papadopoulos 2015).
