# Supplementary material for: The Electoral Coalition of the Radical Right in Western Europe
Source: Br J Sociol. 2025 Dec 7;77(2):300–17. doi: 10.1111/1468-4446.70062 (PMC12950201; doi:10.1111/1468-4446.70062)
Supplement: Supplementary file 1 — Supporting Information S1 [file BJOS-77-300-s001.docx]

# 8. Appendix

**Table A1:** Overview of the items used to operationalise the three different dimensions of political conflict.

|  | **Redistribution** | **Cultural Liberalism** | **Migration** |
| --- | --- | --- | --- |
| **ESS 8** | The government should take  measures to reduce differences in income levels.  Large differences in people’s  incomes are acceptable to  properly reward differences in talents and efforts.  For a society to be fair, differences in people’s standard of living should be small. | Gay men and lesbians should be free to live their own life as they wish.  If a close family member was a gay man or a lesbian, I would feel ashamed.  Gay male and lesbian couples should have the same rights to  adopt children as straight  couples. | Would you say it is generally bad or good for [country]’s economy that people come to live here from other countries?  Would you say that [country]’s  cultural life is generally under-mined or enriched by people coming to live here from other countries?  Is [country] made a worse or a better place to live by people coming to live here from other countries? |
| **ESS 9** | The government should take measures to reduce differences  in income levels.  A society is fair when income and wealth are Equally distributed among all people. | Gay men and lesbians should be free to live their own life as they wish.  If a close family member  was a gay man or a lesbian, I would feel ashamed.  Gay male and lesbian couples should have the same rights to  adopt children as straight couples. | Would you say it is generally bad or good for [country]’s economy that people come to live here from other countries?  Would you say that [country]’s  cultural life is generally undermined or enriched by people coming to live here from other countries?  Is [country] made a worse or a better place to live by people coming to live here from other countries? |
| **ESS 10** | How important do you think it is for democracy in general that the government takes measures to reduce differences in income levels?  The government should take measures to reduce differences in income levels. | Gay men and lesbians should be free to live their own life as they wish.  If a close family member was a gay man or a lesbian, I would feel ashamed.  Gay male and lesbian couples should have the same rights to  adopt children as straight  couples. | Would you say it is generally bad or good for [country]’s economy that people come to live here from other countries?  Would you say that [country]’s cultural life is generally undermined or enriched by people coming to live here from other countries?  Is [country] made a worse or a better place to live by people coming to live here from other countries? |

**Table A2:** Results of factor analyses with voters of the radical right

ESS 8 (1,267 Observations)

| Variable | **Migration** | **Cultural**  **Liberalism** | **Redistri- bution** | Uniqueness |
| --- | --- | --- | --- | --- |
| The government should take  measures to reduce differences in income levels. (reverse-coded) | -0.018 | -0.017 | **0.596** | 0.644 |
| Large differences in people’s incomes are acceptable to properly reward differences in talents and efforts. | 0.031 | -0.029 | **0.562** | 0.682 |
| For a society to be fair, differences in people’s standard of living should be small. (reverse-coded) | 0.044 | -0.036 | **0.596** | 0.642 |
| Would you say it is generally bad or good for [country]’s economy that people come to live here from other countries? | **0.720** | 0.015 | -0.024 | 0.482 |
| Would you say that [country]’s cultural life is generally undermined or enriched by people coming to live here from other countries? | **0.765** | 0.063 | 0.057 | 0.407 |
| Is [country] made a worse or a better place to live by people coming to live here from other countries? | **0.795** | 0.119 | -0.002 | 0.354 |
| Gay men and lesbians should be free to live their own life as they wish. (reverse-coded) | 0.090 | **0.719** | -0.053 | 0.472 |
| If a close family member was a gay man or a lesbian, I would feel ashamed. | 0.062 | **0.629** | -0.019 | 0.600 |
| Gay male and lesbian couples should have the same rights to adopt children as straight couples. | -0.133 | **-0.586** | -0.030 | 0.639 |
|  | | | | |

Factor rotation matrix

ESS 9 (1,643 Observations)

| Variable | **Migration** | **Cultural**  **Liberalism** | **Redistri- bution** | Uniqueness |
| --- | --- | --- | --- | --- |
| The government should take measures to reduce differences in income levels. (reverse-coded) | -0.055 | 0.026 | **0.558** | 0.685 |
| A society is fair when income and wealth are Equally distributed among all people. (reverse-coded) | -0.043 | -0.053 | **0.560** | 0.682 |
| Would you say it is generally bad or good for [country]’s economy that people come to live here from other countries? | **0.726** | 0.053 | -0.038 | 0.469 |
| Would you say that [country]’s cultural life is generally undermined or enriched by people coming to live here from other countries? | **0.761** | 0.113 | 0.005 | 0.408 |
| Is [country] made a worse or a better place to live by people coming to live here from other countries? | **0.779** | 0.116 | -0.045 | 0.377 |
| Gay men and lesbians should be free to live their own life as they wish. (reverse-coded) | 0.115 | **0.725** | -0.008 | 0.461 |
| If a close family member was a gay man or a lesbian, I would feel ashamed. | 0.113 | **0.632** | -0.081 | 0.582 |
| Gay male and lesbian couples should have the same rights to adopt children as straight couples. | -0.124 | **-0.629** | -0.060 | 0.585 |
|  | | | | |

Factor rotation matrix

ESS 10 (1,652 Observations)

| Variable | **Migration** | **Cultural**  **Liberalism** | **Redistri- bution** | Uniqueness |
| --- | --- | --- | --- | --- |
| The government should take measures to reduce differences in income levels. | -0.012 | 0.054 | **0.760** | 0.419 |
| How important do you think it is for democracy in general that the government takes measures to reduce differences in income levels? | 0.091 | 0.043 | **0.759** | 0.414 |
| Would you say it is generally bad or good for [country]’s economy that people come to live here from other countries? | -0.080 | **-0.743** | -0.031 | 0.441 |
| Would you say that [country]’s cultural life is generally undermined or enriched by people coming to live here from other countries? | 0.157 | **0.631** | 0.008 | 0.577 |
| Is [country] made a worse or a better place to live by people coming to live here from other countries? | 0.094 | **0.666** | 0.116 | 0.534 |
| Gay men and lesbians should be free to live their own life as they wish. (reverse-coded) | **0.709** | 0.004 | 0.004 | 0.497 |
| If a close family member was a gay man or a lesbian, I would feel ashamed. | **0.760** | 0.155 | 0.055 | 0.395 |
| Gay male and lesbian couples should have the same rights to adopt children as straight couples. | **0.784** | 0.098 | 0.041 | 0.375 |
|  | | | | |

Factor rotation matrix

**Table A3:** Overview of Calinski-Harabasz values for different cluster solutions

| **Number of Clusters** | **Calinski-Harabasz pseudo-F** |
| --- | --- |
| 2 | 1462.210 |
| 3 | 1657.580 |
| 4 | 1765.190 |
| 5 | 1620.090 |
| 6 | 1507.190 |
| 7 | 1466.820 |
| 8 | 1422.510 |
| 9 | 1373.670 |
| 10 | 1314.440 |
| 11 | 1286.930 |
| 12 | 1255.310 |
| 13 | 1241.240 |

**Table A4:** Factor analysis including attitudes towards climate change with data from the ESS 8.

| Variable | **Migration** | **Cultural Liberalism** | **Redistri-bution** | **Climate Change** | Uniqueness |
| --- | --- | --- | --- | --- | --- |
| The government should take measures to reduce differences in income levels. (reverse-coded) | -0.023 | -0.018 | **0.570** | 0.063 | 0.671 |
| Large differences in people’s incomes are acceptable to properly reward differences in talents and efforts. | -0.005 | -0.008 | **0.483** | 0.045 | 0.764 |
| For a society to be fair, differences in people’s standard of living should be small. (reverse-coded) | 0.029 | -0.020 | **0.549** | 0.047 | 0.695 |
| Gay men and lesbians should be free to live their own life as they wish. (reverse-coded) | 0.198 | **0.749** | -0.021 | 0.060 | 0.395 |
| If a close family member was a gay man or a lesbian, I would feel ashamed. | 0.184 | **0.682** | -0.028 | 0.090 | 0.492 |
| Gay male and lesbian couples should have the same rights to adopt children as straight couples. | -0.254 | **-0.663** | -0.028 | -0.059 | 0.492 |
| Would you say it is generally bad or good for [country]’s economy that people come to live here from other countries? | **0.766** | 0.132 | -0.026 | 0.053 | 0.393 |
| Would you say that [country]’s cultural life is generally undermined or enriched by people coming to live here from other countries? | **0.797** | 0.197 | 0.025 | 0.079 | 0.319 |
| Is [country] made a worse or a better place to live by people coming to live here from other countries? | **0.814** | 0.158 | 0.000 | 0.043 | 0.310 |
| To what extent do you feel a personal responsibility to try to reduce climate change? | 0.221 | 0.182 | 0.030 | **0.542** | 0.623 |
| How worried are you about climate change? | 0.084 | 0.107 | 0.105 | **0.542** | 0.676 |
|  | | | | | |

**Table A5:** Factor analysis including attitudes towards climate change with data from the ESS 10

| Variable | **Migration** | **Cultural Liberalism** | **Redistri-bution** | **Climate Change** | Uniqueness |
| --- | --- | --- | --- | --- | --- |
| The government should take measures to reduce differences in income levels. | -0.077 | -0.035 | 0.820 | -0.020 | 0.320 |
| How important do you think it is for democracy in general that the government takes measures to reduce differences in income levels? | -0.087 | -0.018 | 0.820 | -0.006 | 0.320 |
| Gay men and lesbians should be free to live their own life as they wish. (reverse-coded) | -0.191 | -0.771 | 0.027 | -0.082 | 0.361 |
| If a close family member was a gay man or a lesbian, I would feel ashamed. | 0.179 | 0.701 | -0.023 | 0.070 | 0.471 |
| Gay male and lesbian couples should have the same rights to adopt children as straight couples. | 0.231 | 0.715 | -0.037 | 0.102 | 0.423 |
| Would you say it is generally bad or good for [country]’s economy that people come to live here from other countries? | 0.746 | 0.167 | -0.090 | 0.089 | 0.400 |
| Would you say it is generally bad or good for [country]’s economy that people come to live here from other countries? | 0.805 | 0.207 | -0.057 | 0.093 | 0.298 |
| Would you say that [country]’s cultural life is generally undermined or enriched by people coming to live here from other countries? | 0.812 | 0.150 | -0.086 | 0.062 | 0.306 |
| To what extent do you feel a personal responsibility to try to reduce climate change? | 0.231 | 0.220 | -0.079 | 0.564 | 0.573 |
| How worried are you about climate change? | 0.128 | 0.117 | 0.028 | 0.564 | 0.651 |
|  | | | | | |

**Table A6:** Factor analysis with different indicators for cultural liberalism using data from the ESS 8.

| Variable | **Migration** | **Cultural Liberalism** | **Redistribution** | Uniqueness |
| --- | --- | --- | --- | --- |
| The government should take measures to reduce differences in income levels. (reverse-coded) | 0.002 | 0.012 | 0.570 | **0.675** |
| Large differences in people’s incomes are acceptable to properly reward differences in talents and efforts. | 0.028 | 0.069 | 0.520 | **0.724** |
| For a society to be fair, differences in people’s standard of living should be small. (reverse-coded) | -0.057 | 0.021 | -0.557 | **0.686** |
| Would you say it is generally bad or good for [country]’s economy that people come to live here from other countries? | **0.776** | 0.043 | -0.011 | 0.395 |
| Would you say that [country]’s cultural life is generally undermined or enriched by people coming to live here from other countries? | **0.803** | 0.124 | 0.045 | 0.338 |
| Is [country] made a worse or a better place to live by people coming to live here from other countries? | **0.827** | 0.090 | 0.010 | 0.307 |
| If a close family member was a gay man or a lesbian, I would feel ashamed. | 0.159 | **0.570** | 0.004 | 0.650 |
| Gay male and lesbian couples should have the same rights to adopt children as straight couples. | -0.278 | **-0.522** | -0.044 | 0.649 |
| Now I will briefly describe some people. Please listen to each description and tell me how much each person is or is not like you. He believes that  people should do what they're told. He thinks people should follow rules at all times, even  when no-one is watching. | 0.095 | **0.298** | 0.027 | 0.901 |
| Now I will briefly describe some people. Please listen to each description and tell me how much each person is or is not like you. Tradition is  important to him. He tries to follow the customs  handed down by his religion or his family. | 0.127 | **0.345** | 0.000 | 0.865 |
| When jobs are scarce, men should have more  right to a job than women | 0.179 | **0.447** | 0.015 | 0.768 |
|  | | | | |

**Table A7:** Factor analysis including attitudes towards the deservingness of social welfare recipients with data from the ESS 8

| Variable | **Migration** | **Cultural Liberalism** | **Redistri-bution** | **Deserving-ness** | Uniqueness |
| --- | --- | --- | --- | --- | --- |
| The government should take measures to reduce differences in income levels. (reverse-coded) | -0.021 | -0.012 | **0.569** | -0.004 | 0.676 |
| Large differences in people’s incomes are acceptable to properly reward differences in talents and efforts. | -0.014 | -0.011 | **0.475** | 0.158 | 0.749 |
| For a society to be fair, differences in people’s standard of living should be small. (reverse-coded) | 0.028 | -0.019 | **0.547** | 0.028 | 0.699 |
| Would you say it is generally bad or good for [country]’s economy that people come to live here from other countries? | **0.766** | 0.138 | -0.027 | 0.050 | 0.391 |
| Would you say that [country]’s cultural life is generally undermined or enriched by people coming to live here from other countries? | **0.798** | 0.203 | 0.026 | 0.065 | 0.317 |
| Is [country] made a worse or a better place to live by people coming to live here from other countries? | **0.814** | 0.159 | -0.001 | 0.051 | 0.309 |
| Gay men and lesbians should be free to live their own life as they wish. (reverse-coded) | 0.200 | **0.754** | -0.019 | 0.003 | 0.391 |
| If a close family member was a gay man or a lesbian, I would feel ashamed. | 0.185 | **0.687** | -0.023 | 0.039 | 0.492 |
| Gay male and lesbian couples should have the same rights to adopt children as straight couples. | -0.255 | **-0.664** | -0.026 | -0.065 | 0.490 |
| Most unemployed people do not really try to find a job. | 0.180 | 0.141 | 0.104 | **0.477** | 0.709 |
| Many people manage to  obtain benefits and services to  which they are not entitled. | 0.201 | 0.019 | 0.029 | **0.478** | 0.729 |
|  | | | | | |

Factor rotation matrix

**Table A8:** Distribution of clusters within parties.

| **Party** | **Economic Right** | **Economic Left** | **Cultural Right** | **Moderates** |
| --- | --- | --- | --- | --- |
| **FPÖ** | **120** 19.6% | **180** 29.5% | **104** 17.0% | **207** 33.9% |
| **VB** | **32** 23.4% | **42** 30.7% | **18** 13.1% | **45** 32.8% |
| **AfD** | **103** 22.0% | **145** 31.0% | **131** 28.0% | **89** 19.0% |
| **SVP** | **150** 34.3% | **75** 17.2% | **72** 16.5% | **140** 32.0% |
| **RN** | **72** 22.4% | **99** 30.7% | **56** 17.4% | **95** 29.5% |
| **UKIP** | **34** 23.1% | **32** 21.8% | **20** 13.6% | **61** 41.5% |
| **DF** | **35** 22.7% | **42** 27.3% | **35** 22.7% | **42** 27.3% |
| **FrP** | **80** 26.4% | **71** 23.4% | **66** 21.8% | **86** 28.4% |
| **SD** | **117** 26.6% | **117** 26.6% | **134** 30.5% | **72** 16.4% |
| **PS** | **95** 21.1% | **98** 21.8% | **85** 18.9% | **172** 38.2% |
| **Vox** | **53** 27.3% | **51** 26.3% | **37** 19.1% | **53** 27.3% |
| **FvD** | **20** 37.0% | **15** 27.8% | **7** 13.0% | **12** 22.2% |
| **PVV** | **55** 23.1% | **70** 29.4% | **34** 14.3% | **79** 33.2% |
| **LN** | **97** 21.9% | **70** 15.8% | **79** 17.8% | **198** 44.6% |
| **FdI** | **40** 25.0% | **31** 19.4% | **18** 11.3% | **71** 44.4% |

**Figure A1:** Coefficient plot showing the effects of different attitude dimensions on the likelihood of voting for different radical right parties; based on logistic regressions controlling for gender, age, city/country, religiosity, income, education and the other two attitudinal dimensions. All analyses include survey weights.

**Figure A2:** Division into different clusters, showing the 2-cluster solution up to the 10-cluster solution.

**Figure A3:** Differences between 4-cluster solutions with and without the K-Means clustering.

**Figure A4:** Differences between 4-cluster solutions with and without Latent Profile Analysis.

**Figure A5:** Differences between 4-cluster solutions across different waves.

**Figure A6:** Differences between 4-cluster solutions across different countries.

**Figure A7:** Coefficient plot showing the effects of income and education on the likelihood of voting for different radical right parties; based on logistic regressions controlling for gender, age, city/country, religiosity. Models in the upper part do not control for political attitudes, models in the lower part do. All analyses include survey weights.

**Figure A8:** Coefficient plot of the influence of political trust, political efficacy and demand for popular sovereignity on cluster membership. Marginal effects based on multinomial regressions, controlling for sex, age, urban/rural, religiosity, income, education, country. Standard errors clustered by country and year. Models include survey weights. As some of the items used are only included in ESS 10, the analysis is limited to this wave. To measure political trust, the survey asked about trust in politicians and parliament. The following items were used to measure political efficacy: 1) How much would you say the political system in [country] allows people like you to have a say in what the government does? 2) And how much would you say that the political system in [country] allows people like you to have an influence on politics? In order to measure the extent to which there is a preference for popular sovereignty, the following items were included. 1) How important do you think it is for democracy in general ...that citizens have the final say on the most important political issues by voting on them directly in referendums? 2) …that the views of ordinary people prevail over the views of the political elite? 3) …that the will of the people cannot be stopped?


**Figure A9:** Coefficient plot showing the effects of different attitude dimensions on the likelihood of voting for the radical right in different countries, based on logistic regressions controlling for gender, age, city/country, religiosity and the other two attitudinal dimensions. All analyses include survey weights.

**Figure A10:** Coefficient plot of the influence of occupational class on cluster membership. Marginal effects based on multinomial regressions, controlling for country and ESS round. Standard errors clustered by country and year. Models include survey weights.

**Figure A11:** Comparison of cluster analyses with and without attitudes towards climate change, based on data from waves 8 and 10 of the ESS.

**Figure A12:** Coefficient plot of the influence of socio-structural characteristics on voting for the radical right, comparing effects in Western Europe and Eastern Europe. Marginal effects based on multinomial regressions, controlling for country and ESS round. Standard errors clustered by country and year. Models include survey weights.

**Figure A13:** Coefficient plot of the influence of socio-structural characteristics on cluster membership, comparing effects from thirteen different countries. Marginal effects based on multinomial regressions, controlling for country and ESS round. Standard errors clustered by country and year. Models include survey weights.
